# Using Recombinase-Aid Amplification Combined with *Pyrococcus furiosus* Argonaute for Rapid Sex Identification in Flamingo (*Phoenicopteridae*)

**DOI:** 10.3390/ani15010007

**Published:** 2024-12-24

**Authors:** Shenluan Tan, Fanwen Zeng, Wanhuan Zhong, Tanzipeng Chen, Xuanjiao Chen, Li Li, Hengxi Wei, Shouquan Zhang

**Affiliations:** 1 State Key Laboratory of Swine and Poultry Breeding Industry, National Engineering Research Center for Breeding Swine Industry, Guangdong Provincial Key Laboratory of Agro-Animal Genomics and Molecular Breeding, College of Animal Science, South China Agricultural University, Guangzhou 510642, China; 18520482191@163.com (S.T.); lili007@scau.edu.cn (L.L.); 2 Guangzhou Zoo & Guangzhou Wildlife Research Center, Guangzhou 510075, China; 13501486783@163.com (F.Z.); zi_jing_0225@foxmail.com (T.C.); 13631363846@139.com (X.C.); 3 Kunming Institute of Zoology, Chinese Academy of Sciences, Kunming 650201, China; zwhanimal@163.com

**Keywords:** flamingo, sex identification, recombinase-aided amplification (RAA), *Pyrococcus furiosus* Argonaute (*Pf*Ago)

## Abstract

Flamingos (*Phoenicopteridae*) are among the oldest birds worldwide and are loved by people for their bright red feathers. In addition, flamingos are sexually monomorphic birds, and distinguishing between males and females is difficult. The polymerase chain reaction (PCR) is widely used for sex identification. However, the PCR method requires a precise thermal cycler in the laboratory and is time-consuming. Therefore, developing a rapid, sensitive, and accurate method to identify the sex of flamingos is crucial. In this study, we established a sex identification system using a recombinase-aided amplification-*Pyrococcus furiosus* Argonaute (RAA-*Pf*Ago) technique for greater flamingo (*Phoenicopterus roseus*). The greater flamingo-RAA-*Pf*Ago system can identify unknown-sex greater flamingos in less than 1 h and can be visualized using a fluorescent detector or blue light. The results showed that optimal RAA-*Pf*Ago conditions could detect 0.6 ng of genomic DNA and effectively differentiate between males and females. Random sample evaluations revealed that the system had a 100% coincidence rate compared with conventional PCR. In conclusion, this study provides a sensitive, specific, and accurate reference method for greater flamingo sexing.

## Simple Summary

Flamingos (*Phoenicopteridae*), known for their red feathers, are challenging to sex due to their similar appearance in males and females. While PCR is a reliable method for sex identification, it requires lab equipment and is time-consuming. To overcome these limitations, researchers developed a rapid, accurate system for greater flamingos (*Phoenicopterus roseus*) using the RAA-*Pf*Ago technique. This method determines sex in less than an hour using fluorescence or blue light detection and can identify 0.6 ng of DNA. Field tests confirmed 100% accuracy compared to PCR. RAA-*Pf*Ago provides a specific, sensitive, and accurate reference method for greater flamingo sexing.

## 1. Introduction

Flamingos (*Phoenicopteridae*) are ancient birds distributed in tropical and subtropical regions whose fossil record was known from the early Miocene referable to modern taxa [1]. In 1963, Miller reported that the American Flamingo (*Phoenicopterus ruber*) has occurred both in the early and late Pleistocene [2]. Flamingos are social waterbirds with large family flocks worldwide [3]. Six species are extant recognized and commonly assigned to two genera, *Phoenicopterus* and *Phoenicoparrus* [4]. Among these species, American Flamingo (*Phoenicopterus ruber*), Chilean Flamingo (*Phoenicopterus chilensis*), Andean Flamingo (*Phoenicoparrus andinus*), and James’s Flamingo (*Phoenicoparrus jamesi*) are distributed throughout the Americas; another two species, Greater Flamingo (*Phoenicopterus roseus*) and Lesser Flamingo (*Phoenicoparrus minor*), live in Asia, Africa, and Europe [3,5,6]. In the wild, flamingos are threatened by pollution and habitat destruction, and their population has declined globally [7,8]. Lesser flamingos are threatened by habitat loss and hunting [9,10,11]. The population of the Makgadikgadi Pans, one of the largest flamingo habitats, decreased dramatically between 1975 and 2008. Currently, according to estimates, only approximately 530,000 flamingos remain [12]. Some studies have reported that captive flamingos have higher reproductive rates than wild flamingos. As the reproductive success of flamingos in captivity is approximately 50%, a previous study suggested that introducing new individuals to the flock and adjusting the sex ratio can increase the reproduction rate [13]. Sex identification in flamingos is essential before their introduction. It provides managers and researchers with sex information on birds, which is crucial for reproductive management and biodiversity conservation. If sex identification is inaccurate, the estimation of the flock structure will be faulty, resulting in disruption of breeding schemes and failure of propagation [14]. However, flamingos are monomorphic birds that are indistinguishable in their external morphology and behavior [15]. Therefore, sex identification based on phenotypic characteristics is not feasible. Several surgical approaches have been used for sexing birds. For example, Li et al. (2015) used an endoscope to examine the gonads [16]. This method is feasible, but it requires experienced personnel and also will damage the cloaca of the birds. Therefore, an endoscope is not suitable for rare birds such as flamingos.

Currently, molecular sexing is ideal in birds, especially in birds that lack sexual dimorphism. In birds, males have two sex chromosomes called the Z chromosomes (ZZ), whereas females have Z and W chromosomes (ZW). Most birds can be sexed by the difference in the length of intron 21 of the highly conserved chromodomain helicase DNA-binding protein 1 (CHD1) gene on the Z and W sex chromosomes [17]. Polymerase chain reaction (PCR) is one of the most effective, sensitive, and accurate detection methods for bird sex identification. The PCR assay uses a single set of primers to amplify homologous fragments of the female-specific CHD1-W gene; the CHD1-Z gene is present in both sexes [18]. However, this method is time consuming and requires precise laboratory equipment. With the rapid development of molecular biology, isothermal amplification technologies have also been developed. RPA/RAA is an emerging isothermal amplification method that can rapidly amplify within 20–30 min. RPA/RAA can be performed at a constant temperature without expensive equipment [19]. Many studies have used RPA/RAA combined with nucleases, such as the Cas nuclease in the CRISPR/Cas system, for nucleic acid detection. With the detection of these nucleases, nucleic acids can be easily identified and effectively cut [20].

*Pyrococcus furiosus* Argonaute (*Pf*Ago) is a species of Argonaute protein that is widely found in prokaryotic biological systems and has a nuclear acid cleavage function. When the guide DNA (gDNA), a 5′ phosphorylated ssDNA, recognizes the complementary nucleic acid sequence, *Pf*Ago is activated. *Pf*Ago rapidly cleaves the phosphodiester bond between the 10th and 11th nucleotide base of the target sequence from the 5′ end. *Pf*Ago has more nucleic acid selection flexibility, as it is not constrained by the protospacer adjacent motif (PAM) of the target sequence compared to CRISPR [21]. Presently, *Pf*Ago-based detection systems have been systematically applied, such as the LAMP-PfAgo method for the detection of aquatic pathogens and the *Pf*Ago ligase chain reaction (PLCR) method for the detection of SARS-CoV-2 and HPV [22,23].

Previous studies have demonstrated that the RPA/RAA-*Pf*Ago system has the potential for rapid detection of viruses. However, their application to the sex identification of birds has not been reported. This study aimed to establish a highly sensitive and specific rapid detection system for greater flamingo sex identification as prototype, which is envisaged to surpass PCR in terms of time cost, sensitivity, and convenience. This innovative technique provides a new method for greater flamingo sex identification and can possibly be extended to other species.

## 2. Materials and Methods

### 2.1. Sample Collection

Fourteen unknown-sex greater flamingos and eight known-sex greater flamingos (four males and four females) were collected from the Guangzhou Zoo, Guangdong Province, China. Feathers were stored in a plastic bag labeled with a numerical code for a specific individual and frozen at −20 °C until use. All animal experimental protocols were reviewed and approved by the Guangzhou Zoo Animal Use and Care Committee. All methods were performed in accordance with the relevant regulations, and the recommendations outlined in the ARRIVE guidelines for conducting research were followed.

### 2.2. DNA Extraction and Conventional PCR

Approximately 5–10 mm segments from two to three individual feathers were cut from the root end and placed in a 1.5 mL Eppendorf tube. Total genomic DNA was extracted using the HiPure Tissue and Blood DNA Kit (D3018; Megan, Guangzhou, China) according to the manufacturer’s instructions. After the extraction, DNA was measured using a Nano-Drop ND-2000 spectrophotometer (ThermoFisher Scientific, Waltham, MA, USA) and stored at −20 °C until needed.

PCR amplification was performed using the 2550F/2718R primers proposed by Fridolfsson and Ellegren [24]. The PCR assay was performed using 2 μL of the DNA solution obtained from feathers in a final volume of 20 μL, containing 10 μL of 2× Dream*Taq* PCR Master Mix polymerase (K1081, ThermoFisher Scientific), 4.0 μL of ddH_2_O, and 1 μL (10 μM) of each 2550F/2718R primer. The amplification protocol was composed of the following steps: initial denaturation at 94 °C for 3 min, followed by 35 cycles of 94 °C denaturation for 30 s, annealing at 49 °C for 45 s, and extension at 72 °C for 1 min, followed by a final extension at 72 °C for 5 min. The PCR amplification products were separated by electrophoresis on a 1.5% agarose gel containing ethidium bromide and visualized using an ultraviolet transilluminator (Bio-Rad). DdH_2_O was used as a negative control.

### 2.3. Design and Selection of RAA Primer

To attain the optimal amplification efficiency of RAA, primers were designed according to the primer design principles of RAA. Based on the sequences of the greater flamingo CHD1 gene on the Z and W chromosomes obtained in our laboratory, four pairs of primers were designed for the CHD1-W gene, but not the CHD1-Z gene, using Primer Premier 5.0 (PREMIER Biosoft). The primer sets were selected using the RAA reaction. All primers were synthesized by Sangon Biotech Co., Ltd. (Shanghai, China) and are listed in Table 1.

### 2.4. Design of ssDNA, gDNA, and Probe

A 161 nt stretch of ssDNA was synthesized in the middle of the RAA-amplified sequence, and seven complementary gDNAs were designed for every two bases. All gDNAs were prepared by phosphorylation using T4 polynucleotide kinase (New England Biolabs, Ipswich, MA, USA). A probe with the highest RAA amplification efficiency was designed within the amplification region of the primer set. The probe was labeled with carboxyfluorescein (FAM) as the fluorophore and Black Hole Quencher-1 (BHQ-1) as the quencher. ssDNA, gDNA, and probes were synthesized by Sangon Biotech Co., Ltd. (Shanghai, China) and are listed in Table 2.

### 2.5. Optimization of RAA Reaction Conditions

The RAA reaction was performed using an RAA nucleic acid amplification kit (Zongche Bio-Sci and Tech Co., Ltd., Hangzhou, China) according to the manufacturer’s instructions. Each reaction system contained one RAA lyophilized powder, 29.4 μL of buffer A, 2 μL of forward primer (10 μM), 2 μL of reverse primer (10 μM), 1 μL DNA template, 13.1 μL of ddH_2_O, and 2.5 μL of buffer B, and then remained at 40 °C for 30 min. Furthermore, to achieve optimal conditions for the RAA reaction, a series of temperatures (37, 38, 39, 40, 41, and 42 °C) and reaction times (10, 15, 20, 30, and 40 min) were tested.

### 2.6. gDNA Selection

Seven gDNAs were selected using the *Pf*Ago cleavage ssDNA reaction. The 20 μL *Pf*Ago reaction contained 2.0 μL of 10× reaction buffer (150 mM Tris/HCl, 1 M NaCl, and pH 8.0), 2.66 mM *Pf*Ago (JiaoHong Biotech, Shanghai, China), 2 μL ssDNA, 1.5 μM gDNA, 5 mM MgSO_4_, and 5 μM probe, and ddH_2_O was added to 20 μL. The reaction was performed in a CFX Connect Real-Time PCR Detection System (Bio-Rad, Hercule, CA, USA) at 95 °C for 30 min, and the FAM fluorescence signal was collected once every 30 s. The products were visualized using a fluorescence detector.

### 2.7. Establishment of RAA- PfAgo Assay

RAA was performed using the previously established parameters. Next, 2 μL of RAA product was added to a 20 μL *Pf*Ago mixture supplemented with 2 μL of 10× reaction buffer (150 mM Tris/HCl, 1 M NaCl, and pH 8.0), 2.66 mM *Pf*Ago (JiaoHong Biotech, Shanghai, China), 150 nM gDNA, 5 mM MgSO_4_, and 500 nM probe, and ddH_2_O was added to 20 μL. The reaction was performed in a CFX Connect Real-Time PCR Detection System (Bio-Rad) at 95 °C for 30 min, and the FAM fluorescence signal was collected once every 30 s. The product was visualized using a fluorescence detector or blue light.

### 2.8. Evaluation of the RAA-PfAgo Assay for Sex Identification

To determine the specificity of the RAA-*Pf*Ago detection system, we used genomic DNA from eight individuals of known sex (four females and four males) as a template performed with the RAA-PfAgo assay described above. To confirm the sensitivity of the RAA-*Pf*Ago detection system, 10-fold serial dilutions of feather sample DNA (60 ng, 6 ng, 0.6 ng, 0.06 ng, 6 pg, 0.6 pg, and 0.06 pg) were used as templates for the RAA reaction, and ddH_2_O was used as the negative control.

### 2.9. Sex Identification Using RAA-PfAgo Assay for Field Test

To validate the effectiveness of this system, 14 unknown-sex greater flamingo feather samples were collected from a zoo, and DNA was extracted from the feathers as described above. DNA was used as the template for both the RAA-*Pf*Ago assay and conventional PCR. The effectiveness of the RAA-*Pf*Ago assay was compared with that of conventional PCR.

## 3. Results

### 3.1. Mechanism of RAA-PfAgo Detection System

**Figure 1:**
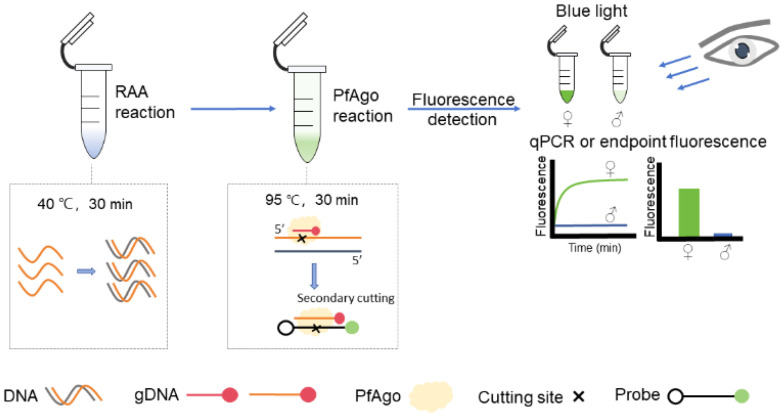
Schematic diagram of RAA-*Pf*Ago assay on greater flamingo sexing. The RAA followed a kit used at 40 °C for 30 min. Then, the RAA amplicons were added to the *Pf*Ago assay and maintained at 95 °C for 30 min. The results were observed via a fluorescence detector or blue light. Positive results appeared, and the fluorescence signal indicated female samples; negative results appeared, and no signal indicated male samples.

Figure 1 shows the greater flamingo sex identification process based on CHD1-W using the RAA-*Pf*Ago method. In the first stage, RAA was amplified specifically for samples containing the CHD1-W gene template. After the activity of *Pf*Ago under the guidance of gDNA, RAA amplification products were first cleaved by *Pf*Ago when the gDNA complementarily paired with the sequence at the cutting site. In the following step, the 5ʹ phosphorylated ssDNA from the first cleavage is a new gDNA that guides *Pf*Ago for secondary cleavage. The probe was used as a cutting substrate in the secondary cleavage, which was labeled with FAM as a fluorophore and BHQ-1 as a quencher. The *Pf*Ago, led by the new gDNA, cleaved the cutting site, and the probe released the FAM fluorophore. Finally, the fluorescence signal was observed using blue light or detected using a fluorescence detector.

### 3.2. RAA Primer Selection

**Figure 2:**
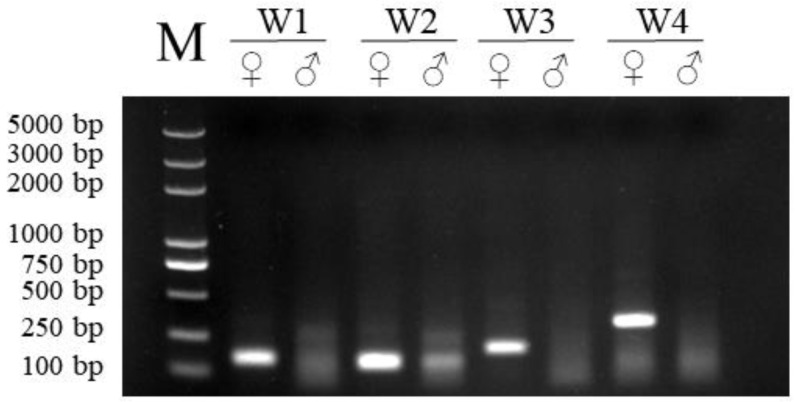
Primer selection using RAA-AGE. The RAA products, by the basic RAA kit with the four primer sets, were subjected to electrophoresis on a 1.5% agarose gel.

The RAA products were subjected to 1.5% gel electrophoresis to verify the size and specificity of the RAA primers (Figure 2). The size of amplified fragments from two (W3 and W4) different primer sets met both predictions and did not contain primer dimers, indicating good specificity for CHD1. At the same template concentration, W4 showed a higher amplification efficiency than the other primers. Based on the above results, W4 was chosen as the best primer for the CHD1 RAA.

### 3.3. Optimization of RAA Conditions

**Figure 3:**
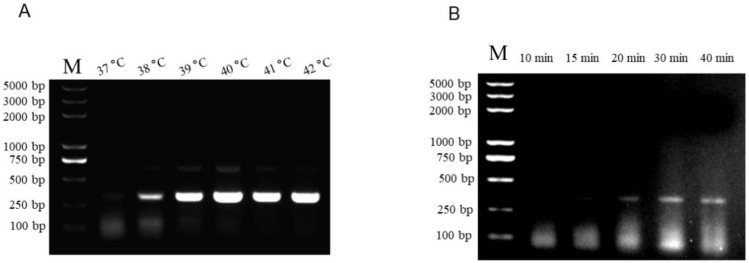
Optimization of the RAA assay. (**A**) Evaluation of different incubation temperatures. (**B**) Evaluation of the effect of different incubation times. The RAA products by the basic RAA kit with the four primer sets were subjected to electrophoresis on a 1.5% agarose gel.

The optimal temperature and time for the RAA reaction were determined using female genomic DNA as a template, and the RAA products were subjected to 1.5% gel electrophoresis. The results (Figure 3A) showed that the lightest fragment appeared at 40 °C; thus, 40 °C was chosen as the optimal temperature of RAA. To determine the optimal time for RAA, the results (Figure 3B) showed that the quantities of amplification products at 30 and 40 min were similar but less than those at 20 min. Finally, 40 °C and 30 min were selected as the optimal conditions for RAA.

### 3.4. gDNA Selection Result

**Figure 4:**
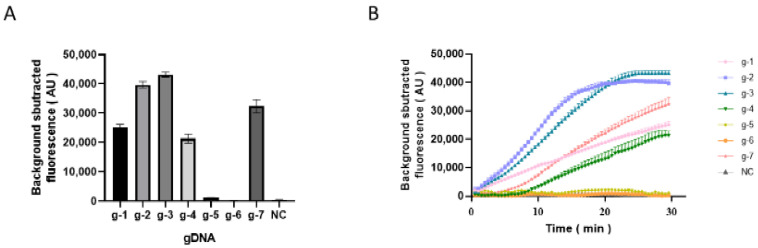
gDNA selection. (**A**) Bar chart illustrating the endpoint fluorescence values of different gDNA. (**B**) Line chart illustrating temporal variation in fluorescence intensity for different gDNA.

The synthesized 161 nt long ssDNA synthesized from the W4 primer set amplification fragment was used as the target sequence. Subsequently, a 16 nt long complementary 5′ phosphorylated gDNA was designed for every two bases. The results of the seven designed gDNA selections by the *Pf*Ago cleavage ssDNA reaction are shown in Figure 4A, B. g-1, g-2, g-3 and g-7 successfully guided *Pf*Ago to cleave ssDNA, and fluorescence values of g-2 and g-3 were similar and higher than g-1 and g-7 (Figure 4A). According to the fluorescence curve, g-2 was more efficient and fastest to reach the plateau (Figure 4B). Therefore, g-2 was selected as the optimal gDNA for subsequent experiments.

### 3.5. RAA-PfAgo Assay Result

**Figure 5:**
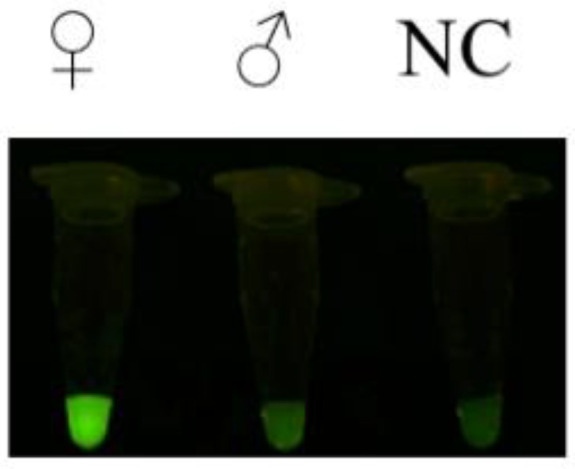
Establishment of the RAA-*Pf*Ago assay. ♀: Female; ♂: Male; NC: ddH_2_O.

One advantage of the greater flamingo sex identification RAA-*Pf*Ago system is that the results can be visualized using a fluorescence detector or under blue light. We used female genomic DNA, male genomic DNA, and ddH_2_O for RAA amplification at 40 °C for 30 min, and then we continued the *Pf*Ago reaction at 95 °C for 30 min. The results under blue light illumination revealed a distinct green fluorescence in female, starkly contrasting with the male (Figure 5), and the female sample did not cross with males.

### 3.6. Optimization of RAA-PfAgo Assay

**Figure 6:**
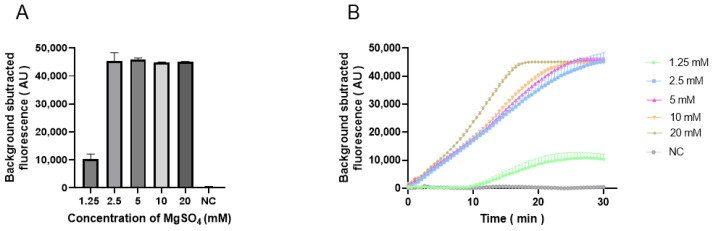
Optimization of Mg^2+^ concentrations (**A**) Bar chart illustrating the endpoint fluorescence values at different concentrations of Mg^2+^. (**B**) Line chart illustrating temporal variation in fluorescence intensity for different concentrations of Mg^2+^.

*Pf*Ago functions efficiently in divalent cation-mediated systems. Mg^2+^ was used as the divalent cation in this system, and its concentration was optimized. The concentrations of Mg^2+^ were set to 1.25, 2.5, 5, 10, and 20 mM. No significant difference was observed in the Mg^2+^ concentration from 2.5 to 20 mM; the fluorescence value of 1.25 mM was the lowest (Figure 6A,B). Figure 6B shows that 5 mM Mg^2+^ was more efficient than 2.5 Mm. Therefore, 5 mM Mg^2+^ was determined as the optimal concentration for the RAA-*Pf*Ago assay.

**Figure 7:**
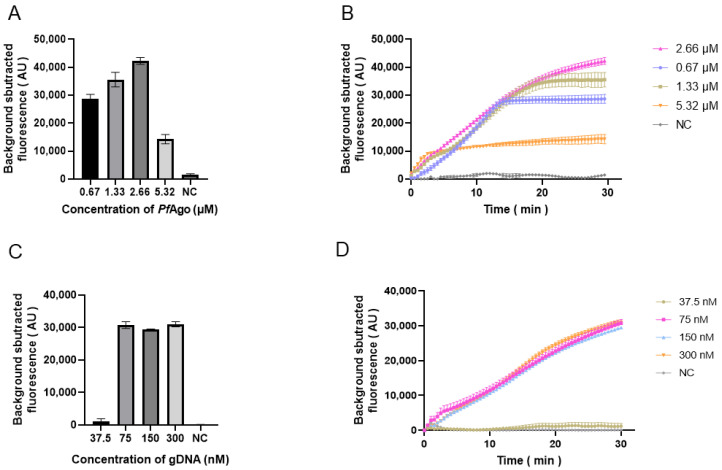
Optimization of *Pf*Ago and gDNA concentrations. (**A**,**C**) Bar charts illustrating endpoint fluorescence values for different concentrations of *Pf*Ago and gDNA. (**B**,**D**) Line charts illustrating the temporal variation in fluorescence intensity at different concentrations of *Pf*Ago and gDNA.

A range of *Pf*Ago concentrations from 0.67 to 5.32 μM (0.67, 1.33, 2.66, and 5.32 μM) were attempted. With an increase in *Pf*Ago, the efficiency of the reaction improved but showed a decreasing trend when the concentration of *Pf*Ago reached 2.66 μM (Figure 7A,B). The highest cleavage efficiency occurred between 1.33 and 2.66 μM. By observing the fluorescence values, 2.66 μM was chosen as the optimal concentration of *Pf*Ago in our system. Four concentrations of gDNA ranging from 37.5 to 300 nM (37.5, 75, 150, and 300 nM) were tested. The fluorescence value was similar between 75 and 300 nM. However, according to the fluorescence curve, the time to reach the plateau was also similar to the increase in gDNA (Figure 7C,D). When the concentration of gDNA was 75 nM, no change was observed; therefore, 75 nM was chosen.

### 3.7. Sensitivity and Specificity of RAA-PfAgo Assay

**Figure 8:**
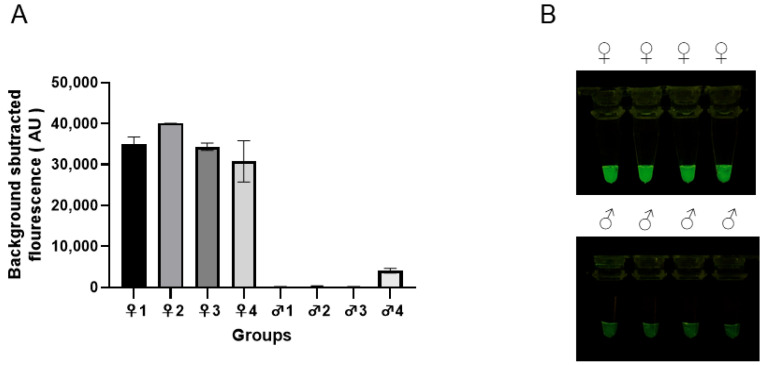
Specificity evaluation of RAA-*Pf*Ago detected both female and male samples. (**A**) Endpoint fluorescence values for RAA-*Pf*Ago sensitivity. (**B**) Observations under blue light.

Eight known-sex greater flamingo genomic DNA were selected as templates to evaluate the specificity of the RAA-*Pf*Ago system. The results observed using the fluorescence detector (Figure 8A) demonstrated that there was a distinct fluorescence signal difference between females and males. Only the female genomic DNA samples show a fluorescence signal. The results (Figure 8B) observed under blue light were same. Thus, the RAA-*Pf*Ago greater flamingo sex identification system has a high specificity.

**Figure 9:**
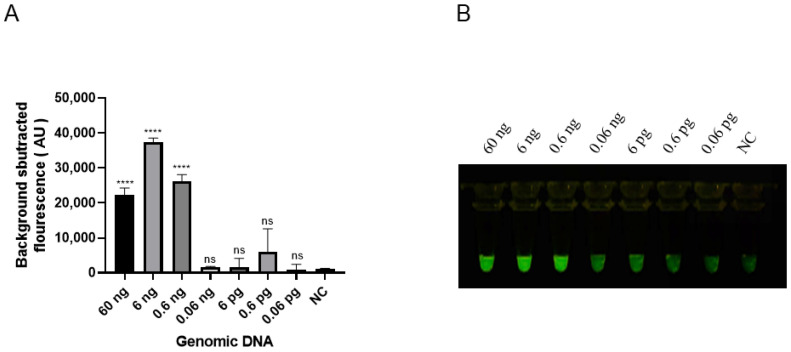
Sensitivity evaluation of RAA-*Pf*Ago. (**A**) Endpoint fluorescence values for RAA-*Pf*Ago sensitivity. (**B**) Results under blue light. NC: negative control. Error bars represent SEM; n = 3; **** *p* < 0.0001.

The sensitivity of the RAA-*Pf*Ago system was tested using a 10-fold serial dilution of genomic DNA ranging from 60 ng to 0.06 pg of the female sample. The fluorescence signal under blue light appeared from 60 ng to 0.6 ng (Figure 9A,B). No fluorescence signal was detected when the genomic DNA was less than 0.06 ng. Therefore, the limit of the RAA-*Pf*Ago greater flamingo sex identification system was 0.6 ng.

### 3.8. Field Sample Test of RAA-PfAgo Assay

**Figure 10:**
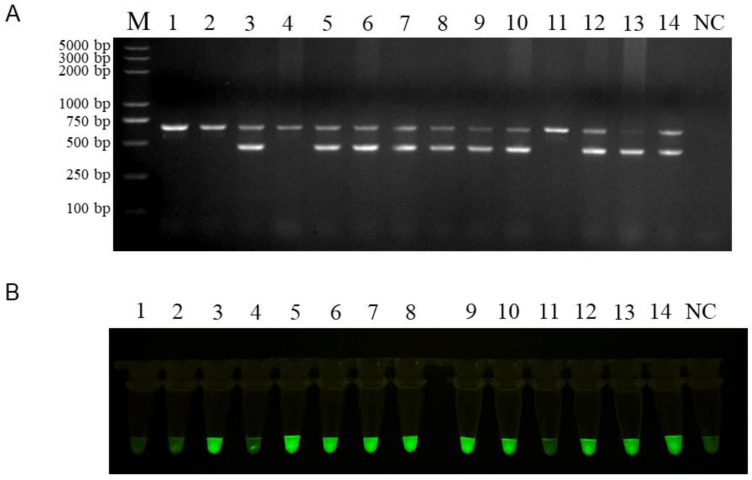
Field tests using conventional PCR and RAA-*Pf*Ago. (**A**) Field samples were tested using conventional PCR on a 1.5% agarose gel. (**B**) The same samples were tested using RAA-*Pf*Ago and visualized under blue light. NC: negative control.

To confirm the validity and reliability of RAA-*Pf*Ago, we performed a parallel test using RAA-*Pf*Ago and conventional PCR on genomic DNA samples from 14 great flamingos of unknown sex. Samples 1, 2, 4, and 11 were detected as male greater flamingos, and samples 3, 5, 6, 7, 8, 9, 10, 12, 13, and 14 were detected as female greater flamingos by RAA-*Pf*Ago, and these results were completely consistent with conventional PCR results (Figure 10A,B). Therefore, RAA-*Pf*Ago is a reliable method for the sex identification of greater flamingos.

## 4. Discussion

With urbanization, industrialization, and the destruction of birds’ habitats, the number of birds in the wild has decreased dramatically [25,26]. An example from the greater flamingo illustrated that a huge population decline was observed during 2019–2021 [10].Therefore, restoring bird populations through conservation and reproduction is important. Some studies have reported that the introduction of new individuals can increase the number of flamingos [27]. Sex identification before introduction is indispensable for flamingos to gain a comprehensive understanding of their population structure and sex ratios. However, flamingos are sexually monomorphic birds, and identifying males and females directly from their appearance is difficult [28]. Molecular biology methods are considered among the most effective methods. In 1996, Griffiths et al. suggested that the CHD1 gene is a sex-linked gene on the W and Z chromosomes of birds and is highly conserved [17]. Therefore, the CHD1 gene was widely used for bird sex identification. Many studies have reported using special primers like P2/P8, 2550F/2718R to amplify homologous fragments of the CHD1 gene on the W and Z chromosomes that are sexed by different lengths [24,29]. PCR is a commonly used molecular method. However, conventional PCR requires precise thermal cycling equipment in the laboratory. The isothermal amplification technique can solve this problem because it can be operated at a constant temperature. LAMP and RAA have recently been used in bird sex identification [30,31,32]. Compare to the LAMP (60–65 °C), the RAA (37–42 °C) reaction temperature is much lower. Furthermore, the LAMP assay requires four to six primers. Owing to the diversity of LAMP amplification products, false-positive results can easily be produced. However, the RAA assay requires only one pair of primers to amplify linear DNA, which is easily detectable and less prone to false positives.

The manual recommends the RAA reaction temperature of 37–39 °C; however, some studies have tested the reaction temperature at 34–44 °C [33]. In this study, temperatures of 37, 38, 39, 40, 41, and 42 °C were tested. According to the results, the band from 37 to 40 °C gradually brightened, and the band was brightest when the temperature reached 40 °C. Lai et al. tested reaction temperatures at 34, 37, 40, and 43 °C for the identification of pigeons’ gender using RPA-LFD. Similar to our results, the strongest test line appeared between 37 and 40 °C and became weaker when the temperature exceeded 40 °C [32]. Another similar result was observed for Wu et al. (2021), who used RAA to detect the African swine fever virus (ASFV) and classical swine fever virus (CSFV) in pigs [34]. The brightest band appeared between 35 and 40 °C, and 39 °C was the brightest. While detection targets vary, the optimal temperature has always been reported to be approximately 40 °C, which is possibly associated with the sensitivity of the primer set. Moreover, time ranges of 10, 15, 20, 30, and 40 min were tested for the optimization of the RAA conditions. Some studies have found that 15 min is the optimal amplification time [35]. Regarding the results, no band appeared before 20 min, the band lightened gradually from 20 min, and no significant change was observed after 30 min. This may be because of the lower concentration of primers, and increasing the primer quantity is a feasible way to shorten the reaction time.

Several methods exist for detecting RAA amplicons, such as LFD. Nuclease-based detection platforms have been widely used for the detection of bacteria, viruses, and parasites [36]. Cas nucleases from the CRISPR system have been used in combination with RAA [37]. *Pf*Ago is another desirable choice that has already been combined with an RAA used in human respiratory syncytial virus (HRSV) and *Salmonella* spp detection [38,39]. *Pf*Ago flexibly functions by the guidance of one 16 nt length 5′-phosphorylated gDNA. In CRISPR, guide RNAs are longer and need complex designs. The simplicity in guide design makes *Pf*Ago potentially more user-friendly, especially for high-throughput applications. PAM sequence is a short DNA sequence located immediately adjacent to the target DNA sequence and required by CRISPR for target recognition and cleavage. The lack of a PAM requirement in *Pf*Ago can be a significant advantage in scenarios where flexibility in target selection and ease of guide design are essential [40,41]. However, the RAA-*Pf*Ago system has not been reported for bird sex identification, and this study attempted to apply the system to this field.

*Pf*Ago has high cleavage capability under divalent cation conditions; Mn^2+^ and Co^2+^ have been used in *Pf*Ago reactions [42]. In this study, Mg^2+^ was used in *Pf*Ago. As the Mg^2+^ concentration increased, the fluorescence values notably did not change when the concentration reached 2.5 mM, which is similar to the results reported [43]. However, in terms of fluorescence values, 5 mM was more efficient than 2.5 Mm. Some studies have reported that when the quantity of divalent cations exceeds a certain value, the reaction exhibits a downward trend [44]. This was not observed in this study. Some studies have reported that different numbers of gDNAs may affect the cleavage efficiency of *Pf*Ago [45,46]. However, no significant differences were observed in endpoint fluorescence values when one, two, or three gDNAs were used. Therefore, only one gDNA sample was used in the present study.

The CHD1 is the most commonly used target for bird sexing. Because the sequence of greater flamingos has not been published, we previously obtained the CHD1 gene sequence on the W and Z chromosomes in the laboratory. Specificity tests showed that only the female samples showed positive results and had no cross-reactivity with the male samples. This demonstrates that this method has high specificity.

In terms of collecting samples from farm animals, such as pigeons, blood can be directly collectively from the wings or toes, resulting in a large amount of DNA being extracted [32]. Fecal samples can also present sensitive and specific results for bird sex identification [47]. In this study, feather samples are minimally invasive for greater flamingo sex identification. DNA was extracted from feather roots following the procedure described above. We set a range of concentration of genomic DNA from 60 ng to 0.06 pg and found that 0.6 ng is the limit. The RAA-*Pf*Ago assay used in this study showed high sensitivity. Conventional PCR and RAA-*Pf*Ago were performed simultaneously to determine the reliability and accuracy of RAA-*Pf*Ago. In the conventional PCR method, primers 2550F/2718R produced approximately 700 bp and 500 bp band. One band appeared in males, and two bands appeared in females. A parallel test between RAA-*Pf*Ago and conventional PCR showed that the 14 field samples were correct, indicating four males and 10 females. The accuracy of RAA-*Pf*Ago in flamingo sex identification was 100%. Sex identification of greater flamingos using RAA-*Pf*Ago was highly accurate. Currently, this method was only conducted on greater flamingos due to the lack of other species materials. In the following study, other species are expected to be used with this method.

## 5. Conclusions

In this study, proper primers, gDNA, and probe design, as well as optimal conditions, enabled the RAA-*Pf*Ago method to efficiently and visually identify the sex of greater flamingos. This method can save approximately 1 h with the same accuracy and specificity of the results compared to the conventional PCR method. Many studies have applied RAA-*Pf*Ago to detect pathogenic microorganisms in humans and animals. To the best of our knowledge, this study is the first to use RAA-*Pf*Ago for greater flamingo sex identification and is expected to be applied to sex identification in other species.

## Figures and Tables

**Table 1:** Primer sequences used in this study.

Primers	Sequences (5′–3′)	Fragment Size (bp)
2550F	GTTACTGATTCGTCTACGAGA	500 and 700
2718R	ATTGAAATGATCCAGTGCTTG	
W1	GAAGTGTTACATTACTCTTATTTCCCTCCC	161
	CAGTGCTTGTTTCCTCAATTCCCCTTTTAT	
W2	GAAGTGTTACATTACTCTTATTTCCCTCCC	151
	TTCCTCAATTCCCCTTTTATTGATCCGTCA	
W3	AAGAATTTTGCTGGTAGTAACCAAGAAGCC	206
	CAATTGGGGAGGGAAATAAGAGTAATGTAA	
W4	AGTTTCCCTTTCAGGTAAGAATTTTGCTGG	338
	TTCCTCAATTCCCCTTTTATTGATCCGTCA	

**Table 2:** ssDNA, gDNA, and probe sequences used in this study.

Name	Sequences (5′–3′)
ssDNA	GAAGTGTTACATTACTCTTATTTCCCTCCCCAATTGTTTTGGCAATTGAG AATTCCAGTTGCTCCGATTAGAATATAGTAGGAGTTCCTTTTTAACTGTA TTATTCAATCTCTTTAGAGACTTGACGGATCAATAAAAGGGGAATTGAG GAAACAAGCACTG
g-1	GGAACTCCTACTATAT
g-2	AACTCCTACTATATTC
g-3	CTCCTACTATATTCTA
g-4	CCTACTATATTCTAAT
g-5	TACTATATTCTAATCG
g-6	CTATATTCTAATCGGA
g-7	ATATTCTAATCGGAGC
Probe	FAM-TAAAAAGGAACTCCTACTATATTCTAAT-BHQ1

## Data Availability

All relevant data are included in this paper. The datasets generated during and/or analyzed during the current study are available from the corresponding author upon request.
